# Conditional diffusion model for inverse prediction of process parameters and dendritic microstructures from mechanical properties

**DOI:** 10.1038/s41598-025-22942-y

**Published:** 2025-10-23

**Authors:** Arisa Ikeda, Ryo Higuchi, Tomohiro Yokozeki, Katsuhiro Endo, Yuta Kojima, Misato Suzuki, Mayu Muramatsu

**Affiliations:** 1 Graduate School of Science and Technology, Keio University, 3-14-1, Hiyoshi, Kohoku-ku, Yokohama, Kanagawa 223-8522 Japan; 2 Department of Aeronautics and Astronautics, The University of Tokyo, 7-3-1, Hongo, Bunkyo-ku, Tokyo, 113-8656 Japan; 3 National Institute of Advanced Industrial Science and Technology, 1-1-1, Umezono, Tsukuba, Ibaraki 305-8568 Japan; 4 Department of Mechanical Engineering, Keio University, 3-14-1, Hiyoshi, Kohoku-ku, Yokohama, Kanagawa 223-8522 Japan

**Keywords:** Mechanical engineering, Mechanical properties, Computational methods

## Abstract

In this study, we develop a conditional diffusion model that proposes the optimal process parameters and predicts the microstructure for the desired mechanical properties. In materials development, it is costly to try many samples with different parameters in experiments and numerical simulations. The use of data-driven inverse design method can reduce the cost of materials development. This study develops an inverse analysis model that predicts process parameters and microstructures. This method can be used for any material, but in this study it is applied to polymeric material, which is the matrix resin of carbon fiber reinforced thermoplastics as an example. Matrix resins contain a mixture of dendrites, which are crystalline phases, and amorphous phases even after crystal growth is complete, and it is important to consider the microstructures consisting of the crystalline structure and the remaining amorphous phase to achieve the desired mechanical properties. Typically, the temperature during forming affects the microstructures, which in turn affect the macroscopic mechanical properties. The trained diffusion model can propose not only the processing temperature but also the microstructure when Young’s modulus and Poisson’s ratio are given. The capability of our conditional diffusion model to represent complex dendrites is also noteworthy. This model can be applied to other process parameters and mechanical properties. Furthermore, multiple process parameters and mechanical properties can be handled together.

## Introduction

In materials development, experiments require trial and error with multiple parameters, which is costly. To solve this problem, numerical simulations have been studied in various fields. In order to accelerate the computation by numerical simulation, hardware performance-based approaches such as parallel computing using graphics processing units parallelism^1^ and software-based approaches using machine learning^2^ have been used. For example, machine learning is used to predict mechanical properties^3–7^. These precious numerical simulations with machine learning are limited to forward analyses. Since forward analysis cannot infer the causes from effects, a data-driven approach to solve the inverse problem is required for more efficient material development. Hashemi et al.^8^ presented a supervised machine learning based computational methodology for the design of particulate multifunctional composite materials with desired thermal conductivity, using the design of particulate composites with liquid metal elastomer as a case study. The method consists of three phases: data generation in the first phase, discovery of complex relationship between the structure and properties using appropriate machine learning algorithms in the second phase, and inference of direct structure-property relationships and generation and visualization of candidate microstructures by inverse design framework in the third phase. This new supervised machine learning approach accelerates the prediction of the thermal conductivity of particle composites and enables the design of composites with desirable properties. Lee et al.^9^ aimed to provide a solution for inverse design even when data quality does not meet high standards, and presented a new design strategy employing two independent approaches: a metaheuristic-assisted inverse reading of conventional forward machine learning models and an atypical inverse machine learning model based on a modified variational autoencoder. They pinpointed several novel thermo-mechanically controlled processed steel alloy candidates, which were validated by a rule-based thermodynamic calculation tool. Bastek et al.^10^ developed a method for the inverse design of nonlinear mechanical metamaterials using a video denoising diffusion model. The video denoising diffusion model is trained on full-field data of periodic stochastic cellular structures. They showed that the model can predict and tune the nonlinear deformation and stress response under compression in the large-strain regime. Hiraide et al.^11^ developed a framework for forward analysis to predict Young’s modulus from the phase-separated structure of polymer alloys and for inverse analysis to predict the structure from Young’s modulus. Forward analysis uses convolutional neural networks (CNNs). For inverse analysis, random search is applied to the combined generative adversarial network (GAN) and CNN model. Moreover, Hiraide et al.^12^ proposed a framework for designing material structures based on macroscopic properties. They use the results of the analysis of the two-dimensional phase separation structure of diblock copolymer melts as structural data. The stress data are obtained by the finite element analysis. The framework consists of a deep learning model that generates structures and a model that predicts the physical properties of the structures. It generates structures with desired physical properties using random search. Vlassis and Sun^13^ presented a denoising diffusion algorithm to discover microstructures with nonlinear fine-tuned properties using the open-source mechanical MNIST data set provided in Lejeune^14^. They used a CNN architecture and a denoising diffusion algorithm, where the CNN architecture predicts the hyperelastic energy functional behavior under uniaxial extension, and the denoising diffusion algorithm generates targeted microstructures with desired constitutive responses. In this way, several data-driven approaches for inverse analysis were proposed for materials development.

Carbon fiber reinforced thermosetting plastics (CFRTSs) and carbon fiber reinforced thermoplastics (CFRTPs) have excellent light weight and strength^15^. These materials are increasingly applied to structural components of aircraft and automobiles^16,17^. CFRTSs have the property of stiffening upon heating and not returning to its original state, whereas CFRTPs have the property of stiffening upon cooling and softening again upon heating. CFRTSs have been applied to many structures, but the material and molding costs are high. In addition, it is difficult to recycle CFRTSs once stiffened, which poses a disposal problem^18,19^. On the other hand, CFRTPs have been attracting attention as a sustainable material due to its low molding cost and recyclability. In CFRTPs, the mechanical properties depend not only on the carbon fibers, but also on the thermoplastic resin used as the base material. The thermoplastic resins used in CFRTPs are crystalline resins, such as polyphenylene sulfide (PPS) and polyether ether ketone (PEEK), which have significant rigidity^20,21^. Crystalline and amorphous phases are mixed in thermoplastic resins, and it is important to consider the microstructures consisting of the crystalline structure and the remaining amorphous phase to achieve the desired mechanical properties. As crystallization progresses, thermoplastic resins become stronger and stiffer. This increases the overall strength and stiffness of the composite. On the other hand, the amorphous phase contributes to the ductility of the composite. Therefore, it is important to consider the balance of crystalline and amorphous phase and their arrangement. In this study, we focus on PPS, a thermoplastic resin with low costs. PPS has a mixture of crystalline and amorphous phases even when the crystals are fully grown. It produces dendritic crystals called dendrites during solidification^22^. Process parameters such as temperature during forming and cooling rate affect the microstructure, which in turn affects the macroscopic mechanical properties^23–28^. As process parameters, not only temperature history but also factors such as pressure can influence the microstructure. Thus, a method of predicting the mechanical properties of resins is required.

Higuchi et al.^29^ and Takashima et al.^30^ developed a multiphysics analysis method for crystalline thermoplastic resins that links the forming conditions, the microstructure and the macroscopic mechanical properties. Crystallization analysis was performed by using the phase-field method^31–33^. The homogenization analysis^34^ of PPS, a crystalline thermoplastic resin, was conducted by using the extended finite element method (XFEM)^35–37^. PPS was characterized to identify the parameters necessary for crystallization analysis. The simulation results were validated by comparing them with experimental data. The details of their studies^29,30^ are as follows. They measured properties such as the glass transition temperature, crystallization temperature and melting point by differential scanning calorimetry (DSC). Moreover, they examined the relationship between the processing temperature and the mechanical properties by tensile tests. The phase-field model they developed reproduced various types of nucleation and polycrystal growth. Conventional methods for dendrite simulation divide the analysis domain into cells and discretely assign states to them, e.g., the cellular automata^38–40^ and Monte Carlo^41,42^ methods. The other method represents the interface by connecting discrete points such as the front-tracking method^43,44^. Higuchi et al.^29^ used the phase-field method to describe the crystal growth of dendrites in thermoplastic resins, in terms of the interface movement between two different phases. Then, they performed the homogenization analysis of the obtained microstructures using XFEM to determine the mechanical properties such as Young’s modulus. By comparing the analytical results with the experimental results, it is shown that their proposed method has a certain validity. Their method is limited to forward analysis, and it is not possible to predict the processing temperature or microstructure from the mechanical properties.

Thus, methods of predicting mechanical properties from microstructures and microstructures from mechanical properties have been developed. However, there is no scheme for proposing process parameters and material microstructures related to how materials should be made. If process parameters such as processing temperature and cooling rate are proposed to produce materials with the desired mechanical property, it will be possible to develop materials efficiently by eliminating the need for trial-and-error experiments using different parameters. However, if only the process parameter is proposed, it is impossible to check its validity without an experiment. By proposing the microstructure together with the process parameter, it is possible to verify its validity by numerical analysis. Therefore, it is important to understand the microstructure underlying the process parameters because the microstructure affects the mechanical properties, which in turn are affected by the process parameters.

**Fig. 1:**
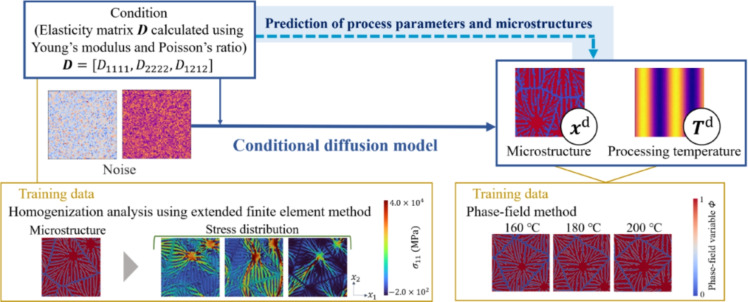
Diagram of conditional diffusion model.

The purpose of this study is to develop a conditional diffusion model that proposes the optimal process parameters and predicts the microstructure on the basis of the desired mechanical properties. In previous studies similar to this study, which developed inverse analysis frameworks, GANs have been applied^11,12,45,46^. In this study, we compared GANs with diffusion models and chose to use diffusion models due to their superior performance. A diffusion model is a new model that can produce high-quality images. Dhariwal et al.^47^ demonstrated that diffusion models provide a good balance between diversity and fidelity. Their ability to handle high-quality images makes them suitable for detailed microstructures, such as the dendrites used in this study. Additionally, GANs require a balance between generators and discriminators, which can result in unstable training. Conversely, diffusion models have the advantages of easy training and no mode collapse. As shown in Fig. 1, the inverse analysis model is applied to thermoplastic resins in this study to suggest the process temperature and predict the microstructure for the desired Young’s modulus and Poisson’s ratio. This model can be applied to other materials, process parameters and mechanical properties by replacing the data used for training. Furthermore, it is possible to handle multiple process parameters and mechanical properties together.

This paper is organized as follows. After describing the details of the methods for crystal growth, homogenization analysis using XFEM and conditional diffusion model, the results and discussion of this study are given. Finally, the conclusions are presented.

## Methods

### Crystal growth

This study introduces two models for the crystal nucleation and growth. The models are used to generate the data of microstructures depending on the temperature. In this study, the crystallization and ambient temperatures are the same owing to the assumption of isothermal forming. Therefore, we consider the crystallization temperature as a parameter that can be specified in the process.

#### Crystal growth by phase-field method

The phase-field method is a method of describing the movement of surfaces by solving the time evolution equation of the phase-field variable $\phi$. In this method, the dimensionless variable $\phi$, which varies between 0 and 1 where $\phi =1$ for the crystalline phase and $\phi =0$ for the amorphous phase, is used to show the spatial distribution of the crystalline and amorphous phases.

The crystal growth is described by the Allen–Cahn and heat conduction equations respectively as follows^33^:

1 $$\begin{aligned} \frac{\partial \phi}{\partial t^\text{p}}=-M^\text{p} \frac{\delta F^\text{p}(\phi )}{\delta \phi}, \end{aligned}$$

2 $$\begin{aligned} \frac{\partial T^\text{p}}{\partial t^\text{p}}=\alpha \nabla ^2T^\text{p}+\frac{\Delta H}{C_\text{p}}\frac{\partial \phi}{\partial t^\text{p}}, \end{aligned}$$

where $\phi$ is the phase-field variable, $t^\text{p}$ is the time, $M^\text{p}$ is the phase-field mobility, $F^\text{p}$ is the free energy of the system, $T^\text{p}$ is the field temperature, $\alpha$ is the thermal diffusivity, $\Delta H$ is the latent heat and $C_\text{p}$ is the specific heat at a constant pressure. The total free energy of the system is expressed as follows^32^:

3 $$\begin{aligned} F^\text{p}(\phi )=\int \left[ f^\text{p}_{\text{doub}}(\phi )+f^\text{p}_{\text{grad}}(\phi )\right] \textrm{d} V, \end{aligned}$$

where $f^\text{p}_\text{doub}$ is the double-well potential and $f^\text{p}_\text{grad}$ is the gradient energy density. Here, $f^\text{p}_\text{doub}$, $f^\text{p}_\text{grad}$, $m^\text{p}(\hat{T}^\text{p})$ and $\hat{T}^\text{p}$ are respectively expressed as

4 $$\begin{aligned} f^\text{p}_{\text{doub}}(\phi )=W \int _0^\phi \phi \left( \frac{1}{2}-\phi -m^\text{p}(\hat{T}^\text{p})\right) (1-\phi ) \textrm{d}\phi , \end{aligned}$$

5 $$\begin{aligned} f^\text{p}_{\text{grad}}(\phi )=\frac{1}{2}\epsilon ^2(\nabla \phi )^2, \end{aligned}$$

6 $$\begin{aligned} m^\text{p}(\hat{T}^\text{p})=\frac{a_\text{k}}{\pi} \arctan (\gamma (1-\hat{T}^\text{p})), \end{aligned}$$

7 $$\begin{aligned} \hat{T}^\text{p}=\frac{T^\text{p}-T^\text{p}_\text{c}}{T^\text{p}_\text{m}-T^\text{p}_\text{c}}, \end{aligned}$$

where *W* is the height of the energy barrier, $\hat{T}^\text{p}$ is the dimensionless temperature and $\epsilon$ is the coefficient of interface energy gradient. The constants are set as $a_\text{k}=0.9$ and $\gamma =10$. From Eq. (7), we find that the crystal growth depends on the crystallization temperature $T^\text{p}_\text{c}$.

#### Formation of crystal nuclei

For the modeling of crystal nucleation, in this study, we introduce the model proposed by Pantani et al.^48^ as

8 $$\begin{aligned} \frac{d N(T^\text{p}(t^\text{p}))}{d t^\text{p}}=N_0 \exp \left[ -\frac{C_1}{\left( T^\text{p}(t^\text{p})-T^\text{p}_{\infty}\right)}\right] \exp \left[ -\frac{C_2\left( T^\text{p}(t^\text{p})+T^\text{p}_\text{m}\right)}{T^\text{p}(t^\text{p})^2\left( T^\text{p}_\text{m}-T^\text{p}(t^\text{p})\right)}\right] , \end{aligned}$$

where $\frac{d N(T^\text{p}(t^\text{p}))}{d t^\text{p}}$ is the nucleation rate, *N* is the nucleation density, $T^\text{p}$ is the field temperature, $t^\text{p}$ is the time, $N_0$ is a constant independent of temperature, $T^\text{p}_{\infty}$ is the temperature at which molecular motion stops completely, $C_1$ and $C_2$ are nucleation rate parameters and $T^\text{p}_\text{m}$ is the melting point obtained at a particular crystallization temperature $T^\text{p}_\text{c}$. The Hoffman–Weeks plot shows the crystallization temperature $T^\text{p}_\text{c}$ on the horizontal axis and the melting point $T^\text{p}_\text{m}$ on the vertical axis. DSC^49^ gives the melting point from the starting temperature of the melting peak of the DSC curve. Thus, nucleation depends on the crystallization temperature $T^\text{p}_\text{c}$. In Eq. (8), ${T^\text{p}_\text{m}}=a{T^\text{p}_\text{c}}+b$, where $T^\text{p}_\text{c}$ is the crystallization temperature (ambient temperature), $a = 0.0948$ is the slope of the approximate line between the crystallization temperature and the melting point obtained from the Hoffman–Weeks plot, and $b = 253.7$ is the intercept of the line^50^. Although this model assumes homogeneous nucleation in which the nucleation occurs stochastically on the basis of nucleation rate, the actual nucleation is heterogeneous because, for example, unknown inclusions can accelerate the nucleation. To consider this effect, a few initial nuclei are initially introduced in this study.

#### Generation of crystal microstructure by the phase-field method

To generate the microstructure of thermoplastic resins for machine learning, we use the phase-field method. The size of a nucleus is equal to one grid for simplicity. The analysis is performed at three crystallization temperatures: 160 °C, 180 °C and 200 °C. These temperatures have been selected because the differences in microstructure are easily recognisable under these conditions. Table 1 shows the conditions used in the analysis by the phase-field method. The condition parameters in the phase-field method are obtained from the references^33,51^.

**Table 1:** Analysis conditions for phase-field method.

Number of calculation steps	20,000
Number of grid points	$320\times 320$
Crystallization temperature $T^{\text{p}}_{\text{c}}$	160 °C, 180 °C, 200 °C
Temperature at maximum crystallization rate $T^\text{p}_{\text{c} \, \textrm{max}}$	180 °C
Glass transition temperature $T^\text{p}_\text{g}$	100 °C
Number of initial nuclei	2
Boundary condition	Periodic boundary condition

### Homogenization analysis using XFEM

#### XFEM

The elasticity matrix $\varvec{D}$ used as the condition of the diffusion model is obtained by homogenization analysis using XFEM for thermoplastic resins generated by the phase-field method. At the interface between the crystalline and amorphous phases, the strain is discontinuous and the local modification of the interpolation function allows accurate approximation. The nodes for XFEM are generated on the basis of the differentially discretized grid for the phase-field method and the triangular elements are generated by dividing the square grid into two parts. In addition, subelements are introduced for elements containing interfaces to integrate discontinuous functions with high accuracy. In XFEM, the displacement is expressed as

9 $$\begin{aligned} \varvec{u}^{\textrm{h}}=\sum _{I=1} N^\text{x}_I(\varvec{x}^\text{x}) (\varvec{u}_I+R(\varvec{x}^\text{x}) \varvec{a}^\text{x}_I), \end{aligned}$$

where *I* is the node, $N^\text{x}_I(\varvec{x}^\text{x})$ is the shape functions, $\varvec{x}^\text{x}$ is the coordinates, $\varvec{u}_I$ is the vector of node displacement, $R(\varvec{x}^\text{x})$ is the enriched function to be introduced locally in the interpolating function and $\varvec{a}^\text{x}_I$ is the nodal degree of freedom for the basis function $N^\text{x}_I(\varvec{x}^\text{x})R(\varvec{x}^\text{x})$ added by the enriched function. The ramp function proposed by Moës et al.^52^, which is introduced as an enriched function, can express the continuity of displacement and its derivative, which is the discontinuity of strain, and is expressed as

10 $$\begin{aligned} R(\varvec{x}^\text{x})=\sum _{I=1} |\psi _I|N^\text{x}_I(\varvec{x}^\text{x})-\left| \sum _{I=1} \psi _I N^\text{x}_I(\varvec{x}^\text{x})\right| , \end{aligned}$$

where $\psi _I$ is the level set function at node *I* and is expressed in terms of the phase-field variable $\phi$ as

11 $$\begin{aligned} \psi =2\phi -1. \end{aligned}$$

Thus, $\psi =0$, which means that the phase-field variable $\phi =0.5$, is recognized as the interface between the crystalline and amorphous phases.

#### Elasticity matrix calculated by homogenization analysis using XFEM

In this study, we use microstructure analysis tools that combine XFEM and the homogenization method^36^. We obtain the stress distribution using XFEM. Then, we obtain the mean stress by the homogenization method assuming a homogeneous body. The key-degree-of-freedom method proposed by Li et al.^53^, which imposes boundary conditions via the additional degree of freedom apart from the model mesh, makes it easier to treat macroscopic stresses and strains. In this study, we use the nonlinear XFEM code combined with phase-field simulation^37^.

**Fig. 2:**
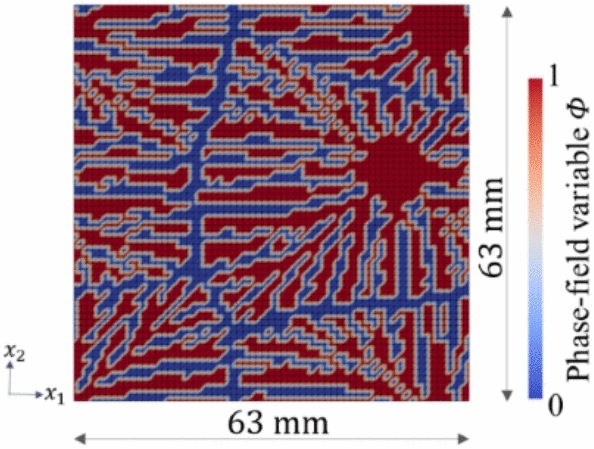
Analysis size of XFEM.

Table 2 shows the conditions for XFEM. Images of microstructures generated by the phase-field method are binarized and compressed and input to XFEM. The mean values of $5 {\ \textrm{pixels}}\times 5 {\ \textrm{pixels}}$ regions are calculated for each $320 {\ \textrm{pixels}}\times 320 {\ \textrm{pixels}}$ analysis grid to be compressed to $64 {\ \textrm{pixels}}\times 64 {\ \textrm{pixels}}$. The phase-field variable, which takes continuous values from 0 to 1, is binarized to 0 and 1 using a threshold of 0.5, both before and after compression. Because stress transfer paths are critical in XFEM, the threshold is chosen to preserve the connectivity of crystal chains, ensuring that they do not disconnect or unintentionally merge with neighboring chains. Therefore, the images input to XFEM are compressed to $64 {\ \textrm{pixels}}\times 64 {\ \textrm{pixels}}$ pixels and binarized to 0 and 1. The validity of compression is examined in the “Dataset” subsection of the Results and Discussion section and in the Supplementary Information, while the validity of binarization is discussed in the Supplementary Information. The analysis size of XFEM is shown in Fig. 2. The length order of this phase-field method is nondimensionalized. Since dimensions are not relevant in homogenization analysis using XFEM, the dimensionless model is directly loaded into a program with an mm-t-s unit system and solved on the mm order. Table 3 shows the physical properties of the PPS used for XFEM. The elasticity matrix $\varvec{D}$ is obtained by the identified Young’s modulus and Poisson’s ratio^29,30^ (Supplementary Information).

**Table 2:** Analysis conditions for XFEM.

Size of analysis area	$63{\ \textrm{mm}}\times 63{\ \textrm{mm}}$
Incremental time	1.0 s
Newton–Raphson method convergence index	0.1
Boundary condition	Periodic boundary condition

**Table 3:** Physical properties of crystalline and amorphous phases in homogenization analysis using XFEM.

	Crystal	Amorphous
Young’s modulus *E*	28,000 MPa^54^	150 MPa^29,30^
Poisson’s ratio $\nu$	0.2^29,30^	0.4^29,30^

### Conditional diffusion model

**Fig. 3:**
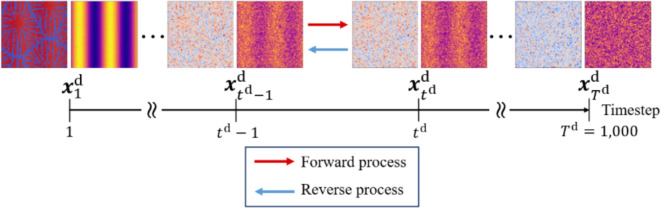
Forward and reverse processes of conditional diffusion model.

**Fig. 4:**
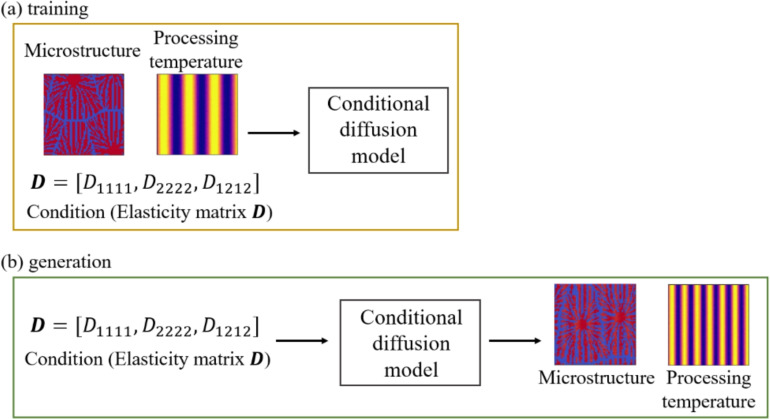
Diagram of (**a**) training and (**b**) generation of conditional diffusion model.

In this study, we employ a diffusion model for predicting the process parameters and the microstructures for the desired mechanical properties. The diffusion model^47,55–59^ was proposed by Sohl-Dickstein et al.^55^ and improved as the denoising diffusion probabilistic model (DDPM) by Ho et al.^56^. The model used in this study is DDPM with condition, which is the called conditional diffusion model. The conditional diffusion model consists of the forward and reverse processes, as shown in Fig. 3. In the forward process, Gaussian noise is added to the images at each timestep using Markov chains. The forward process is defined as follows^56^:

12 $$\begin{aligned} q(\varvec{x}_{1:T^\text{d}}^\text{d}|\varvec{x}_0^\text{d}) = \prod _{t^\text{d}=1}^{T^\text{d}} q\left( \varvec{x}^\text{d}_{t^\text{d}}|\varvec{x}^\text{d}_{t^\text{d}-1}\right) , \end{aligned}$$

13 $$\begin{aligned} q(\varvec{x}_{t^\text{d}}^\text{d}|\varvec{x}^\text{d}_{t^\text{d}-1}) = \mathcal {N}\left( \varvec{x}^\text{d}_{t^\text{d}};\sqrt{1-\beta _{t^\text{d}}}\varvec{x}^\text{d}_{t^\text{d}-1},\beta _{t^\text{d}}\varvec{I}\right) , \end{aligned}$$

where $\varvec{x}^\text{d}_0$ is the data, $\varvec{x}^\text{d}_{T^\text{d}}$ is the noise, $\varvec{x}^\text{d}_{1} - \varvec{x}^\text{d}_{T^\text{d}}$ are the latents, $t^\text{d}$ is the arbitrary timestep, $T^\text{d}$ is the total number of timesteps for noise addition and removal, *q* is the stochastic process of the forward process, $\mathcal {N}$ is the normal distribution, $\beta _1, \ldots ,\beta _{T^\text{d}}$ are the variance schedule, $\sqrt{1-\beta _{t^\text{d}}}\varvec{x}^\text{d}_{t^\text{d}-1}$ is the mean and $\beta _{t^\text{d}}\varvec{I}$ is the variance. In the reverse process, U-Net is used to remove the noise at each timestep. During training, the model learns parameters for generating images from noise by repeatedly adding to and removing noise from images in the forward and reverse processes. The reverse process is defined as follows^56^:

14 $$\begin{aligned} p_\theta (\varvec{x}^\text{d}_{0:{T^\text{d}}}) = p(\varvec{x}^\text{d}_{T^\text{d}}) \prod _{t^\text{d}=1}^{T^\text{d}} p_\theta (\varvec{x}^\text{d}_{t^\text{d}-1}|\varvec{x}^\text{d}_{t^\text{d}}), \end{aligned}$$

15 $$\begin{aligned} p_\theta (\varvec{x}^\text{d}_{t^\text{d}-1}|\varvec{x}^\text{d}_{t^\text{d}}) = \mathcal {N}\left( \varvec{x}^\text{d}_{t^\text{d}-1};\varvec{\mu}_\theta (\varvec{x}^\text{d}_{t^\text{d}},t^\text{d}),\sum \limits _{\theta}(\varvec{x}^\text{d}_{t^\text{d}},t^\text{d})\right) , \end{aligned}$$

where *p* is the stochastic process of the reverse process, $p_\theta$ is the stochastic process of the reverse process using the neural network and $\varvec{\mu}_\theta$ is parameterized as a neural network. As shown in Fig. 4, the training data are the microstructures of thermoplastic resins generated by the phase-field method, the processing temperatures specified in the phase-field analysis and the elasticity matrix $\varvec{D}$ obtained by homogenization analysis using XFEM for the microstructure generated by the phase-field analysis. For the training data of processing temperature, we generate image patterns of processing temperature (IPPT). Note that IPPT does not mean the distribution of temperatures, but gives a pattern for temperature conditions.

**Fig. 5:**
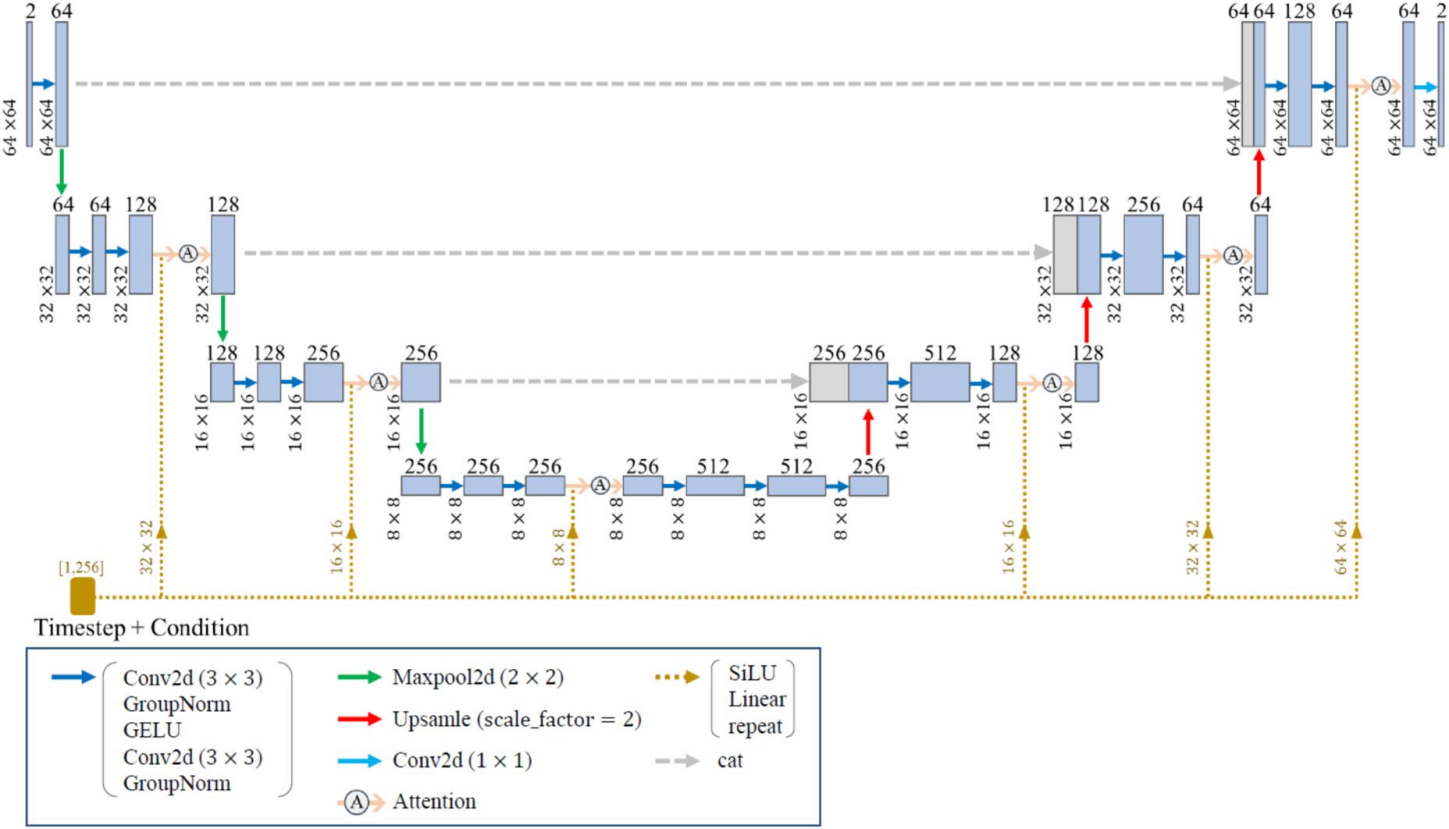
Structure of U-Net.

Noise is added to and removed from images of the microstructures and IPPTs. In the process of image generation, only the reverse process of the diffusion model is used to generate images from noise distributions. We use U-Net to remove the noise at each timestep. The U-Net used in this study is shown in Fig. 5. U-Net is a method developed by Ronneberger et al.^60^ for semantic segmentation, in which features are extracted from original images by an encoder and the obtained images are reconstructed in the same size as the original images by a decoder on the basis of the extracted features. Detailed information such as location information is not lost by maintaining feature maps with skip connections at each process. It is possible to change the method of removing for each condition by considering the information of the timestep in the processes of adding and removing noise. In this study, the component of the elasticity matrix $\varvec{D}$ is used as the condition. In U-Net, the condition and time information are added to the image at each of the encoding and decoding processes, as shown in Fig. 5. Then, the condition and time information are convolved with the image.

The training data are the microstructures of thermoplastic resins, IPPTs and the elasticity matrix $\varvec{D}$. The microstructures are generated by the phase-field method, IPPT means the temperature specified in the phase-field analysis and the elasticity matrix $\varvec{D}$ is obtained by homogenization analysis using XFEM for the microstructures generated by the phase-field method. We employ images of microstructures upon crystal growth completion. Images of the microstructures of dendrites are compressed for use in training machine learning. The images used for machine learning are compressed and binarized in the same manner as those used in XFEM. For input to the machine learning model, the microstructure images are rescaled to binary values of –1 and 1, consistent with the pixel value range of the processing temperature images, which also range –1 to 1. To construct the dataset, it is essential to secure a sufficiently large analysis area to capture the dendritic microstructures observed in experiments. However, due to computational constraints, the image size must be reduced to $64 {\ \textrm{pixels}}\times 64 {\ \textrm{pixels}}$ pixels or smaller. If the resolution is coarsened by conducting phase-field simulations directly at $64 {\ \textrm{pixels}}\times 64 {\ \textrm{pixels}}$ pixels, the resulting analysis area becomes too small to adequately capture crystal growth. Conversely, conducting simulations at higher resolution with a larger domain results in a high compression ratio, potentially causing adjacent crystal chains to merge. Thus, the phase-field method analysis is performed on a $320 {\ \textrm{pixels}}\times 320 {\ \textrm{pixels}}$ area, and the resultant image is compressed to $64 {\ \textrm{pixels}}\times 64 {\ \textrm{pixels}}$. In addition, continuous phase-field variables are binarized before and after compression. Moreover, we perform data augmentation on the microstructures generated by the phase-field method. The $180^\circ$ rotation, inversion with respect to the $x_1$ axis, inversion with respect to the $x_1$ axis and $180^\circ$ rotation are conducted for the same elasticity matrix $\varvec{D}$ value.

**Fig. 6:**
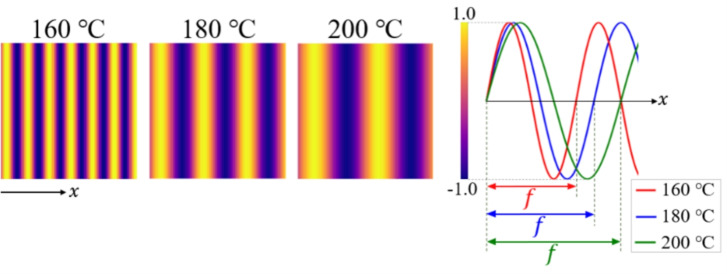
Image pattern of processing temperature (IPPT). Image patterns are produced for each processing temperature, where the frequency *f* is a function of the crystallization temperature $T^\text{p}_\text{c}$ which means the processing temperature.

To represent processing temperatures in the training data, we initially used images in which all pixels had constant values corresponding to each scalar temperature. However, this approach did not result in successful training. Therefore, we adopted images with embedded patterns, which enabled more effective learning. To classify the temperature on the basis of the images, we create different patterns of images of IPPT at each temperature. We specify $f=\frac{2.0}{T^\text{p}_\text{c} - 140} \, \textrm{Hz}$ so that the frequency is a function of the crystallization temperature and $L^\text{x}(x)=A\sin ({2\pi fx})$ to produce the sinusoidal stripe image as IPPT shown in Fig. 6, where $L^\text{x}$ is the luminance, *A* is the amplitude, *f* is the frequency, *x* is the abscissa coordinate and $T^\text{p}_\text{c}$ is the crystallization temperature. While the IPPTs are generated using $L^\text{x}$ as the pixel value, the pixel values are scaled to lie within the range of –1 to 1. The reason for using sinusoidal stripe images as the training data is that these images can be used for determining the temperature through Fourier transform using the determined frequency. The data of microstructures and IPPT are $64 {\ \textrm{pixels}}\times 64 {\ \textrm{pixels}}\times 1 {\ \textrm{channel}}$, for a total of $2 {\ \textrm{channels}}$.

The elasticity matrix $\varvec{D}$ given as the condition is a vector with three components. The conditions used in training are $D_{1111}$, $D_{2222}$ and $D_{1212}$, which are normalized from 0 to 1. For the diffusion model, the time information during noise addition and removal is important. The model is trained by adding 256-dimensionally expanded conditions to 256-dimensionally expanded time information. Specifically, the original three-component condition values are repeated to extend the dimension, and the extra components are filled with zeros. The time information is extended to 256 dimensions by positional encoding. By adding the condition to the time information, we can change the weight of each condition so that the diffusion model learns a different way to remove noise depending on the condition.

Table 4 shows the number and size of data used for training. To avoid overfitting, we stopped training before an upward trend in the loss of the validation data.

**Table 4:** Data used to train $64{\ \textrm{pixels}}\times 64{\ \textrm{pixels}}$.

Number of training data	13,908
Number of validation data	1728
Number of test data	432
Image size	$64{\ \textrm{pixels}}\times 64{\ \textrm{pixels}}$
Minibatch size	15
Number of epochs	1189

## Results and discussion

### Dataset

**Fig. 7:**
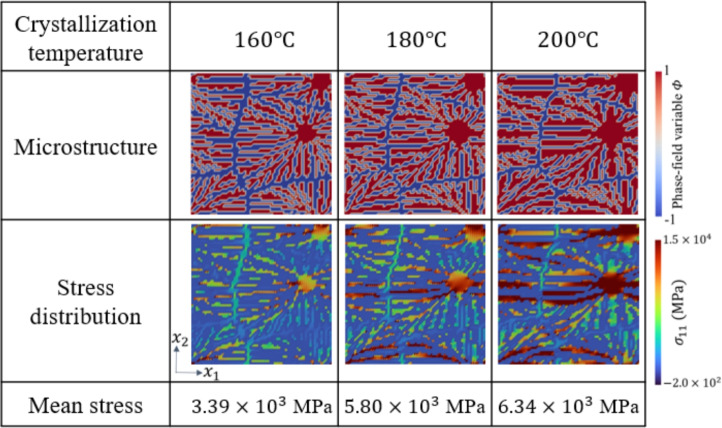
Examples of training data of the microstructures, the distribution of the *x*-directional tensile stress $\sigma _{11}$ and the mean stress.

We discuss the validity of the compression. Since only the phase-field method uses $320 {\ \textrm{pixels}}\times 320 {\ \textrm{pixels}}$ data and the XFEM use $64 {\ \textrm{pixels}}\times 64 {\ \textrm{pixels}}$ data after compression as training data, it is sufficient to confirm that the trend of crystallization temperature derived from the phase-field method is valid. Figure 7 shows examples of training data of the microstructures generated by the phase-field method, the distribution of the *x*-directional tensile stress $\sigma _{11}$ and the mean stress obtained by homogenization analysis using XFEM (under the condition of Eq. (S2a)). The microstructures indicate that the higher the crystallization temperature, the thicker the crystal chains grow. The stress distribution shows that high stresses are generated near the nuclei toward the direction of the imposed strain. Moreover, increasing the crystallization temperature increases the values of stress distribution and mean stress. The trend of thicker crystal chains at higher temperatures is similar to that shown before compression. Therefore, since the trend in the relationship between crystallization temperature and microstructure before and after compression has not changed, compression is not considered problematic.

**Fig. 8:**
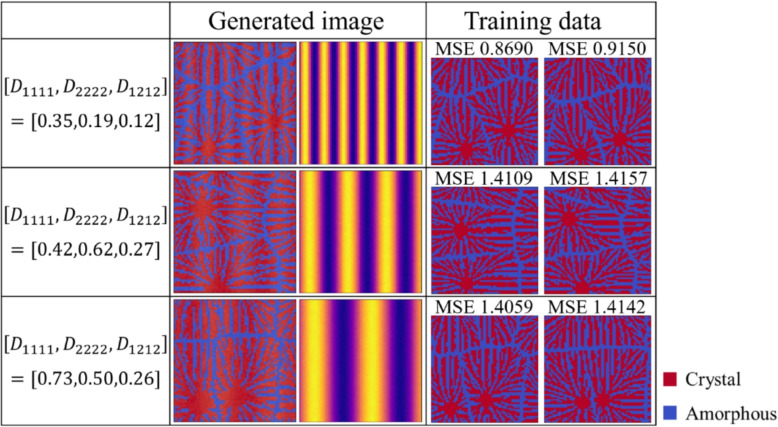
Comparing MSEs of training data and generated images to see if image regurgitation has not occurred.

If the quality of the training data is poor, image regurgitation, which generates images exactly the same as the training data, may occur. In order to confirm that image regurgitation has not occurred, we compare the mean squared errors (MSEs) between the training data and the generated images. The value of MSE should be large because we want to generate data that is different from the training data. As shown in Fig. 8, MSE is sufficiently large to show that the generated microstructure is different from the microstructure used for training.

### Validation of proposed processing temperatures and microstructures

**Fig. 9:**
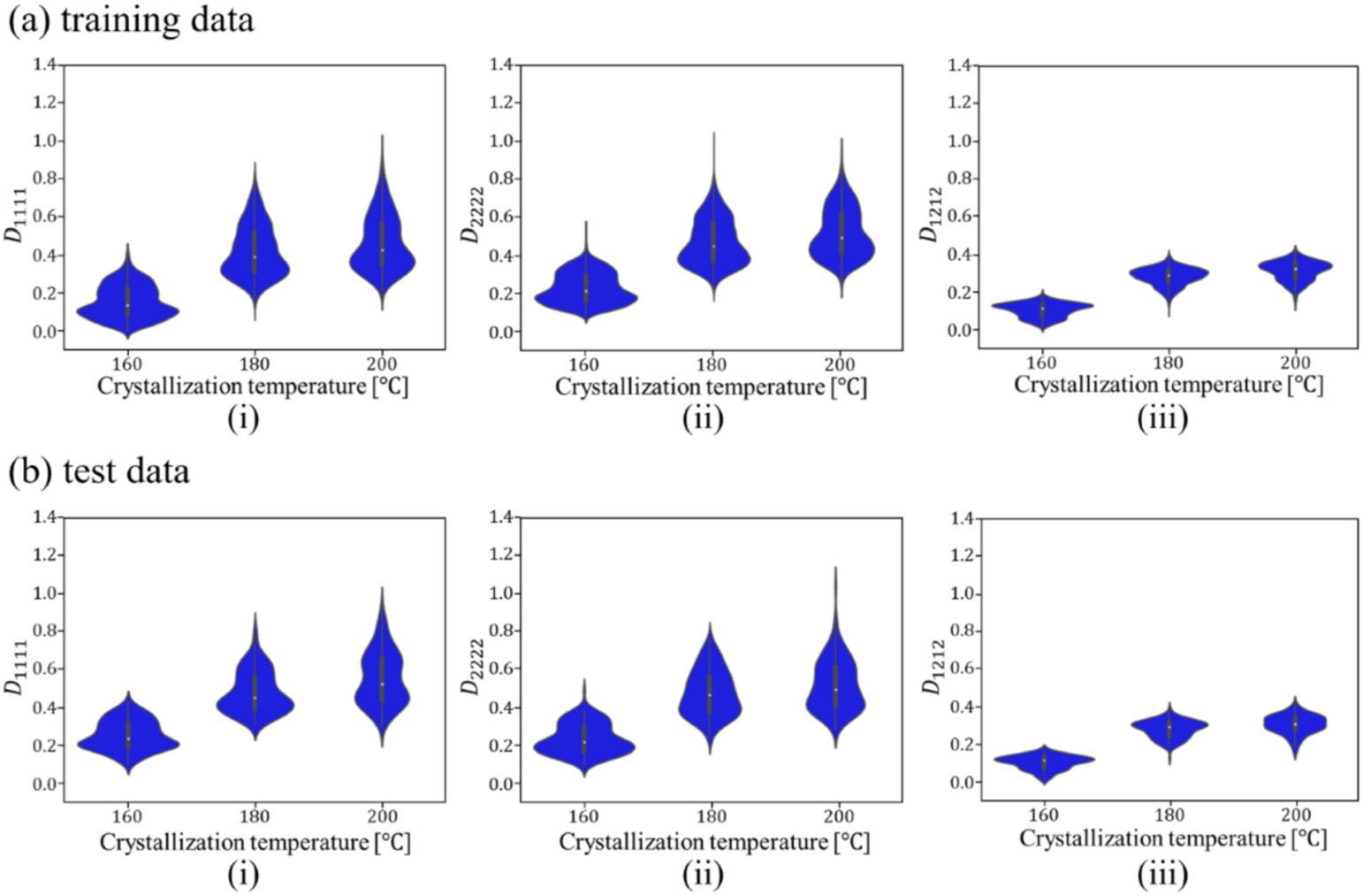
Relationship between crystallization temperature and elasticity matrix (i) $D_{1111}$, (ii) $D_{2222}$ and (iii) $D_{1212}$ in (**a**) training and (**b**) test data.

**Fig. 10:**
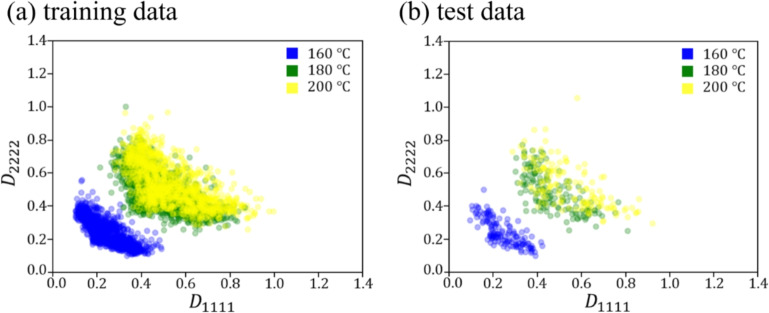
Relationship between $D_{1111}$ and $D_{2222}$ for each crystallization temperature in (**a**) training and (**b**) test data.

Figures 9 and 10 show the relationship between the crystallization temperature specified in the phase-field analysis and the elasticity matrix $\varvec{D}$ obtained by XFEM for the microstructures generated by the phase-field method for (a) training and (b) test data, respectively. The violin plots in Fig. 9 show the distribution of the data with the horizontal axis for the crystallization temperature and the vertical axis for the value of each component of the elasticity matrix $\varvec{D}$. Each violin plot shows that there is a large amount of data in the bulging part of the plot and a large scatter of data in the vertically long part of the plot. The black rectangle in the plot represents the interquartile range of the box plot, and the white dot at the center represents the median value. Fig. 9 shows that $D_{1111}$ and $D_{2222}$ show similar trends, and the higher the crystallization temperature is, the larger the value of the components of the elasticity matrix $\varvec{D}$ tends to be. Similarly, $D_{1212}$ also shows an increasing trend with crystallization temperature. However, its values are smaller than those of the other components, and the scatter of $D_{1212}$ is also smaller. Figure 10 shows the elasticity matrix $\varvec{D}$ obtained from the image of the phase-field method after compression as the input of XFEM. In this figure, scatter plots are shown with $D_{1111}$ on the horizontal axis and $D_{2222}$ on the vertical axis, with different colors indicating the crystallization temperature. It can be seen that the higher the temperature is, the upper the right side of the plot is, and the values of each component of $D_{1111}$ and $D_{2222}$ are larger. These plots show that the higher the crystallization temperature, the larger the value of each component of the elasticity matrix $\varvec{D}$.

**Fig. 11:**
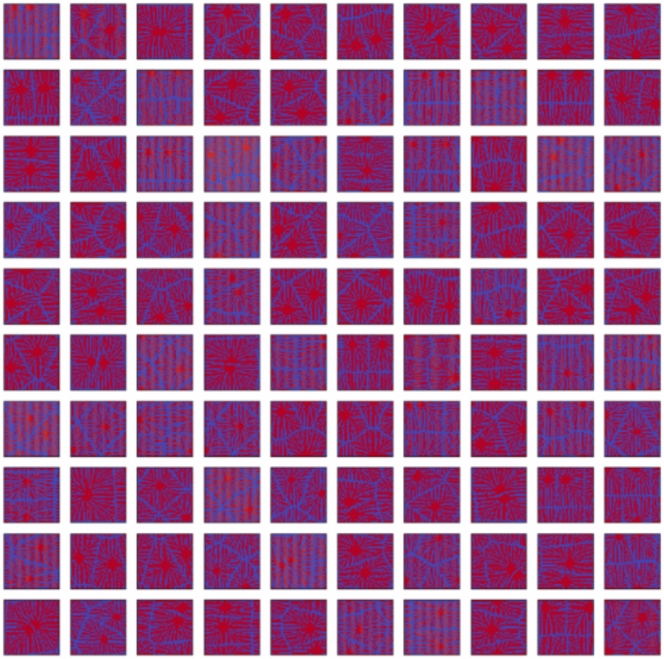
Predicted microstructures generated under conditions of test data.

**Fig. 12:**
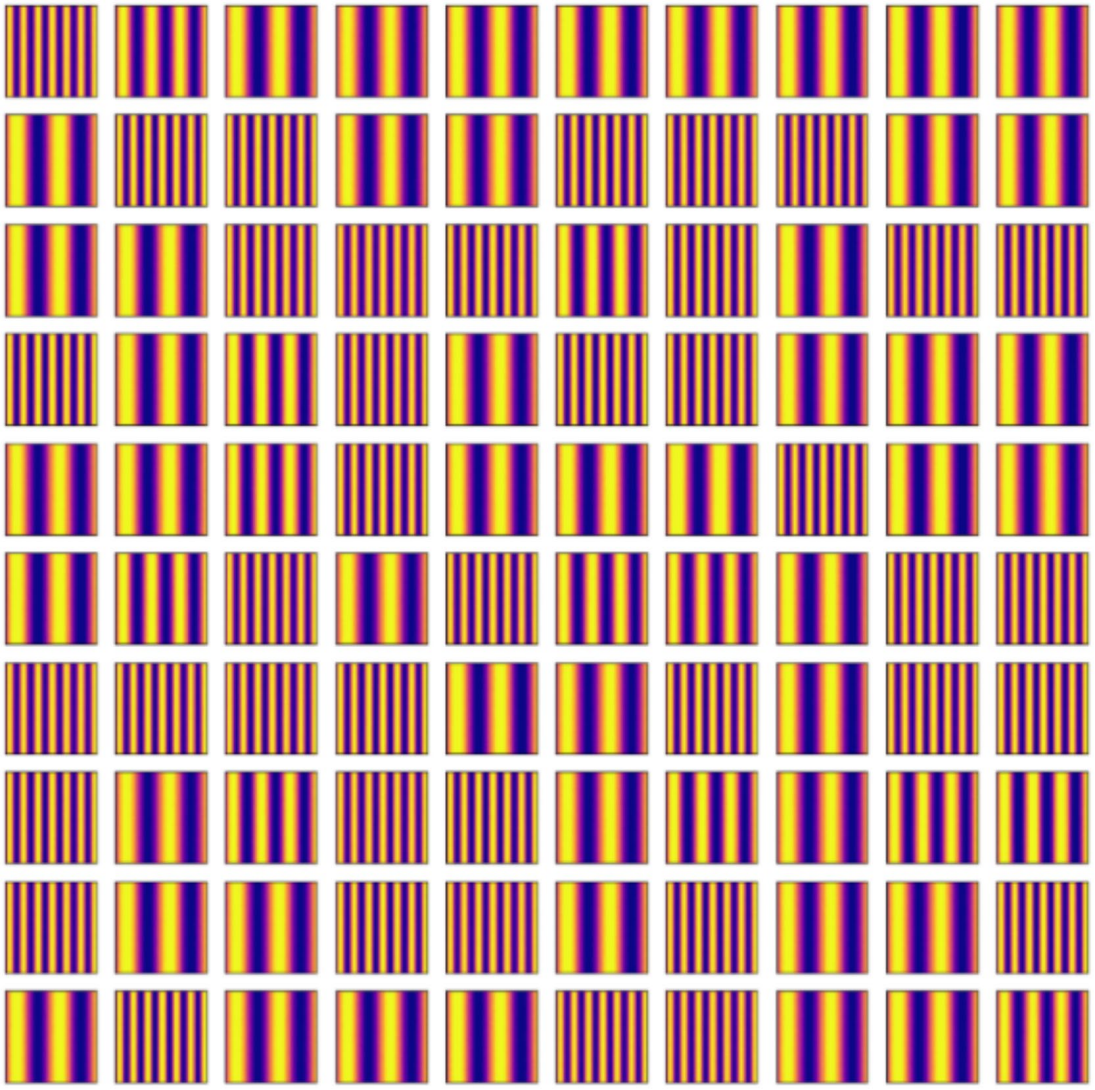
Proposed processing temperatures generated under conditions of test data.

**Fig. 13:**
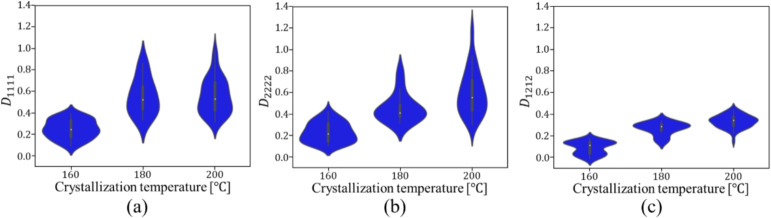
Relationship between crystallization temperature and elasticity matrix (**a**) $D_{1111}$, (**b**) $D_{2222}$ and (**c**) $D_{1212}$ generated under conditions of test data.

**Fig. 14:**
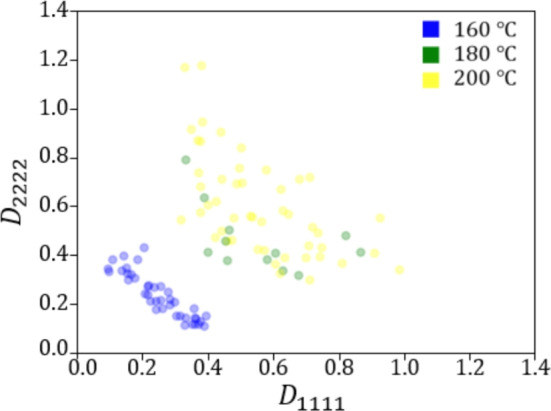
Relationship between $D_{1111}$ and $D_{2222}$ generated under conditions of test data for each crystallization temperature.

**Fig. 15:**
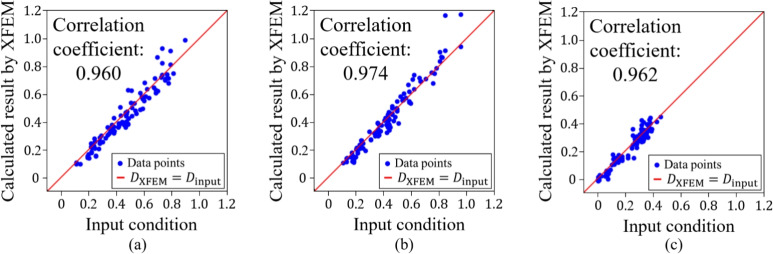
Correlation coefficients of elasticity matrix (**a**) $D_{1111}$, (**b**) $D_{2222}$ and (**c**) $D_{1212}$ generated under conditions of test data.

Firstly, we confirmed the trained model by using the condition of the test data. Figures 11, 12, 13, 14 and 15 show the results. Figure 11 shows the generated images of microstructures and Fig. 12 shows the generated images that indicate the processing temperature. Figure 11 shows an example of the generated microstructures, and various dendrite microstructures could be generated. In addition, as shown in Fig. 12, each microstructure image is associated with a corresponding processing temperature image. Figures 11 and 12 show that the crystal chains are thicker at higher crystallization temperatures and thinner at lower crystallization temperatures, which is similar to the trend observed in the training data. Figure 13 shows the relationship between the proposed temperature and the elasticity matrix $\varvec{D}$ obtained by homogenization analysis using XFEM again for the generated microstructure. Figure 14 is a scatter plot with $D_{1111}$ on the horizontal axis and $D_{2222}$ on the vertical axis, plotted in different colors for different crystallization temperature. From Figs. 13 and 14, the relationship between crystallization temperature and elasticity matrix $\varvec{D}$ is generally appropriate because the tendencies of the training data and test data are similar. Figure 15 compares the values of each component of the elasticity matrix $\varvec{D}$ obtained by homogenization analysis using XFEM of the generated microstructure again with the condition given for generation. The red line indicates the ideal situation that the elasticity matrix $\varvec{D}$ obtained by homogenization analysis using XFEM for the generated microstructure is equal to the elasticity matrix $\varvec{D}$ given as the condition, i.e., when $D_\text{XFEM} = D_\text{input}$. Where $D_\text{XFEM}$ is the element of the elasticity matrix $\varvec{D}$ obtained by homogenization analysis using XFEM of the generated microstructure again, $D_\text{input}$ is the element of the elasticity matrix $\varvec{D}$ given for generation. From the correlation coefficients and each plot, the elasticity matrix $\varvec{D}$ obtained by the homogenization analysis using XFEM shows good agreement with the condition given for generation. The correlation coefficients indicates a strong positive correlation, which is considered that the predicted microstructure is appropriate for the given condition.

### Results for conditions not included in the dataset

**Fig. 16:**
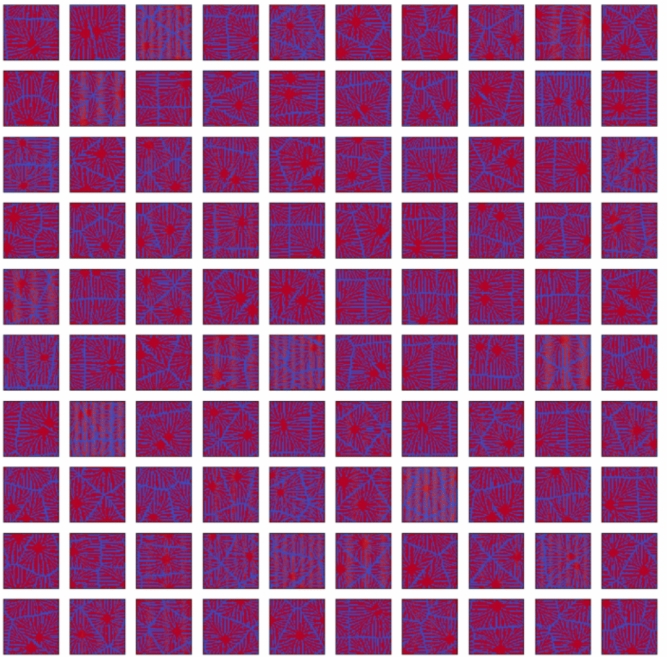
Predicted microstructures generated under the conditions that are not included in the dataset.

**Fig. 17:**
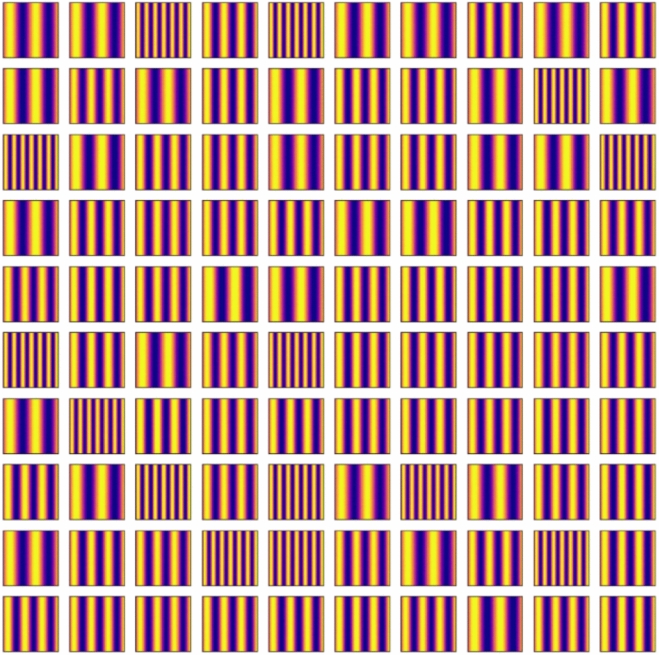
Proposed processing temperatures generated under the conditions that are not included in the dataset.

**Fig. 18:**
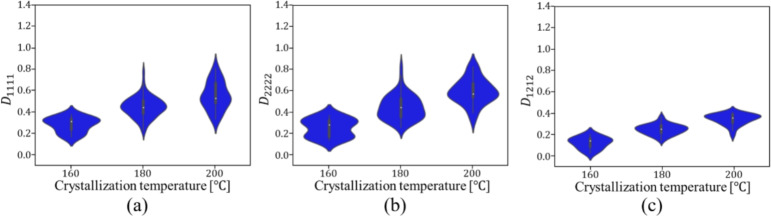
Relationship between crystallization temperature and elasticity matrix (**a**) $D_{1111}$, (**b**) $D_{2222}$ and (**c**) $D_{1212}$ generated under the conditions that are not included in the dataset.

**Fig. 19:**
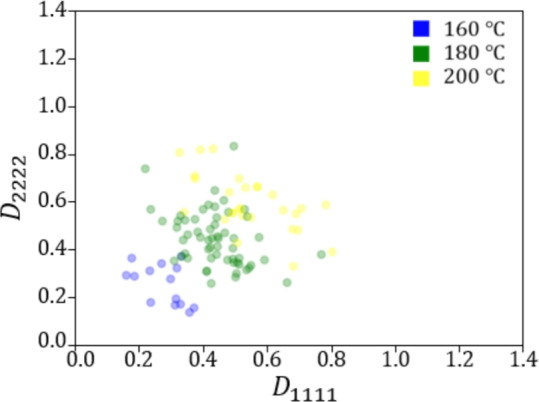
Relationship between $D_{1111}$ and $D_{2222}$ generated under the conditions that are not included in the dataset for each crystallization temperature.

**Fig. 20:**
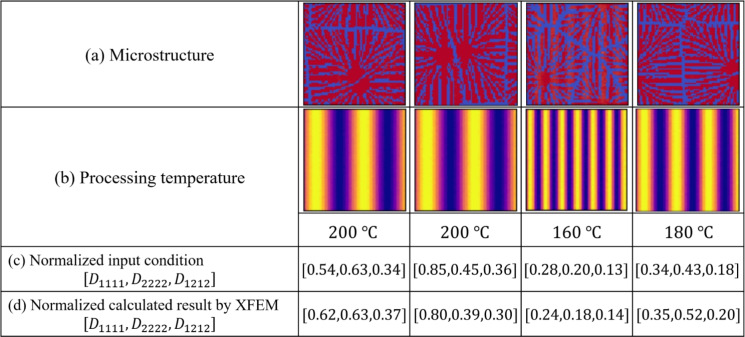
Results of generation under the conditions that are not included in the dataset.

**Fig. 21:**
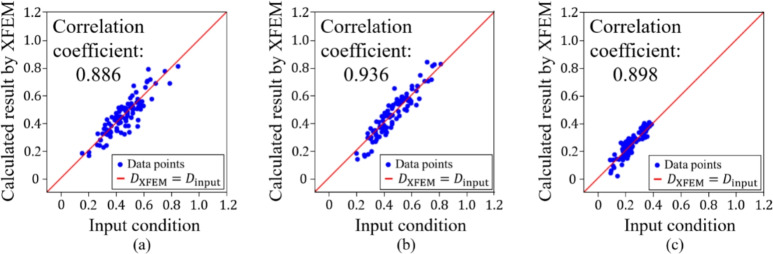
Correlation coefficients of the elasticity matrix (**a**) $D_{1111}$, (**b**) $D_{2222}$ and (**c**) $D_{1212}$ generated under the conditions that are not included in the dataset.

We confirm the results for the conditions not included in the dataset. In contrast to the test data, this is a new condition not obtained by the homogenization analysis using XFEM for the images of the phase-field method. The results are shown in Figs. 16, 17, 18, 19, 20 and 21. Figure 16 shows the generated images of microstructures and Fig. 17 shows the generated images that indicate the processing temperature. As shown in Fig. 16, detailed microstructures of various dendrites can be generated even for conditions that are not included in the data set. In addition, processing temperatures corresponding to the microstructures are generated as images, as shown in Fig. 17. The tendency of thicker crystal chains at higher crystallization temperatures and thinner crystal chains at lower crystallization temperatures is similar to that observed in the training data. Figure 18 shows the relationship between the proposed temperature and the elasticity matrix $\varvec{D}$ obtained by homogenization analysis using XFEM for the generated microstructure. Figure 19 is a scatter plot with $D_{1111}$ on the horizontal axis and $D_{2222}$ on the vertical axis, plotted in different colors for different crystallization temperatures. The higher the crystallization temperature, the upper right of the plot, indicating that the values of $D_{1111}$ and $D_{2222}$ are larger. Figures 18 and 19 show that the relationship between crystallization temperature and the elasticity matrix $\varvec{D}$ is generally similar to that of the training and test data, even when the data are generated using the conditions not included in the dataset. Figure 20 shows examples of the generated results. The model developed outputs images of microstructures and IPPTs. Figure 20 shows (a) the generated microstructures, (b) IPPTs, (c) the conditions and (d) the elasticity matrix $\varvec{D}$ values obtained by homogenization analysis using XFEM for the generated microstructure. When the condition (c) is put into the trained model, the images (a) and (b) are generated. As shown in Fig. 20a, the conditional diffusion model can represent the detailed microstructure of dendrite crystals of thermoplastic resins. By homogenization analysis with XFEM to obtain the elasticity matrix $\varvec{D}$ for the predicted microstructure, we find that elasticity matrix $\varvec{D}$ is close to the condition given for generation. Figure 21 shows the elasticity matrix $\varvec{D}$ for each component. The horizontal axis represents the elasticity matrix $\varvec{D}$ obtained by homogenization analysis using XFEM for the generated microstructure, and the vertical axis is the condition given for generation. The correlation coefficients and each plot indicate that the values of elasticity matrix $\varvec{D}$ obtained by homogenization analysis using XFEM agree with the condition given for generation with good accuracy. The correlation coefficients imply a strong positive correlation between input condition and calculated result by XFEM, suggesting appropriate microstructure for the given condition.

### Demonstration of the proposed conditional diffusion model

**Fig. 22:**
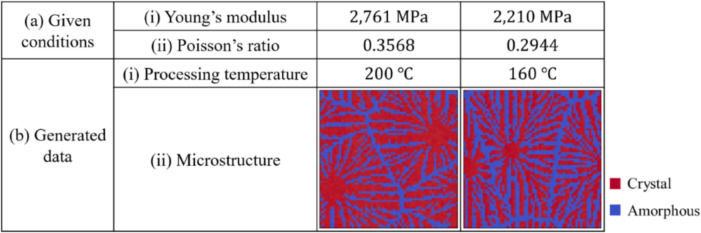
Proposed processing temperatures and predicted microstructures obtained using conditional diffusion model.

The conditional diffusion model developed in this study can propose the optimal processing temperature and predict the microstructure for given Young’s modulus and Poisson’s ratio. The advantage of this model is that it outputs not only the processing temperature but also the microstructure as images. Figure 22 shows example of the model demonstration. Figure 22 shows (a)(i) the specified values of Young’s modulus, (a)(ii) the specified values of Poisson’s ratio, (b)(i) the proposed temperatures and (b)(ii) the predicted microstructures of thermoplastic resins. We train the model with the conditions obtained by homogenization analysis using XFEM on $320 {\ \textrm{pixels}}\times 320 {\ \textrm{pixels}}$ before image compression. Table 5 shows the number and size of data used for training. The relationship between Young’s modulus and Poisson’s ratio and the calculation of the elasticity matrix $\varvec{D}$ is described in the Supplementary Information. As shown in Fig. 22, 200 °C is proposed for a large Young’s modulus of $2761 \,\text{MPa}$, whereas 160 °C is suggested for a small Young’s modulus of $2210 \,\text{MPa}$. Thus, the results show that the temperature tends to increase with Young’s modulus.

This time, we focused on processing temperature as a process parameter, but this approach can be applied to other types of parameters as well. In this study, we examined the temperature history, which is likely to cause problems in manufacturing. In isothermal crystallization, as in this study, the temperature history includes the crystallization temperature. In this study, this crystallization temperature is considered as the processing temperature. On the other hand, in non-isothermal crystallization, the temperature history includes the cooling rate. In addition to temperature history, factors such as pressure can affect polymer crystals. The phase-field model can be extended to various process parameters by adding terms dependent on pressure to the free energy functional^61^. The conditional diffusion model can be extended to various process parameters, provided that datasets can be generated using the phase-field method. It can also handle multiple process parameters together. In this study, one channel is prepared for the process parameter, and images representing the processing temperature are assigned. By creating multiple process parameter images and assigning one channel to each, multiple process parameters can be handled together.

In this study, the elasticity matrix $\varvec{D}$ and mechanical properties determined by it, such as Young’s modulus and Poisson’s ratio, are used as conditions. However, the method is extendable to other mechanical properties as well. For the elastic constants, the three components of the elasticity matrix $\varvec{D}$ are repeated in the model and extended to 256 dimensions. This makes it easy to adjust the number of components in the input. This method supports the simultaneous input of multiple scalar quantities, thereby enabling the simultaneous handling of multiple mechanical properties. In this study, only linear analysis is performed, but by extending the framework to nonlinear analysis, it will become possible to compute mechanical properties such as yield stress and fracture strength. The model proposed in this study allows the input of multiple mechanical properties, such as yield stress and fracture strength.

These can be realized without modifying the machine learning model. In the present case, the analysis domain is small and needs to be compressed for the sake of computational resources. The method proposed in this study can be applied to other models such as the latent diffusion model^62^. The latent diffusion model compresses data such as images into a low-dimensional latent space and applies the diffusion model in that space. This model can be used by replacing the image part of the present method with a latent variable vector. This may allow us to handle larger area of images and may eliminate the need for compression. Thus, the problem of compression can be solved by improving the model to a better one. This is the future work of our research.

**Table 5:** Data used to train $320{\ \textrm{pixels}}\times 320{\ \textrm{pixels}}$.

Number of training data	13,908
Number of validation data	1728
Number of test data	432
Image size	$64{\ \textrm{pixels}}\times 64{\ \textrm{pixels}}$
Minibatch size	5
Number of epoch	549

## Conclusions

In this study, we developed a conditional diffusion model that can propose the optimal processing temperature and predict the microstructure for the desired elastic constants of thermoplastic resins. As a result of using the developed model, the following three were confirmed:

- By training the pattern that indicates the processing temperature together with the images of the microstructure, the model could predict not only the processing temperature but also the microstructure when the elastic constants were given.
- The developed model proposed high temperatures with high Young’s modulus. At high temperatures, the predicted crystal structures were obtained with thicker crystal chains.
- Even when the conditions were not included in the dataset, complicated dendritic patterns whose elastic constants were reasonable were reproduced.

In conclusion, the conditional diffusion model developed in this study can propose the optimal processing temperature and predict the microstructure of thermoplastic resins that satisfies the desired mechanical properties. This study enables us to realize an inverse design that proposes the process parameters related to how the material should be made so as to satisfy the mechanical properties.

This model can be applied to other materials, process parameters and mechanical properties by replacing the data used for training, such as the microstructure, processing temperature and elastic constants. In this case, each process parameter is assigned to one channel. By increasing the number of channels and creating an image for each process parameter, it becomes possible to handle multiple process parameters together. By applying the method proposed in this study to other models, such as the latent diffusion model, it may become possible to handle images with a larger spatial range and eliminate the need for image compression. This is the future work of our research.

## Supplementary Information

Supplementary Information.

## Data Availability

The datasets used and/or analyzed in the study are available from the corresponding author on reasonable request.
