# Supplementary material for: Conditional diffusion model for inverse prediction of process parameters and dendritic microstructures from mechanical properties
Source: Sci Rep. 2025 Oct 23;15:37147. doi: 10.1038/s41598-025-22942-y (PMC12549910; doi:10.1038/s41598-025-22942-y)
Supplement: Supplementary file 1 — Supplementary Information. [file 41598_2025_22942_MOESM1_ESM.pdf]

Supplementary Materials

Homogenization analysis and elasticity matrix

The input data for homogenization analysis using XFEM are phase-field variables, grid information, material properties and boundary conditions. The relationship between stress and strain is expressed as

$$\left\{ \begin{array}{c} \sigma_{11} \\ \sigma_{22} \\ \tau_{12} \end{array} \right\} = \left[\begin{array}{ccc} D_{1111} & D_{1122} & D_{1112} \\ D_{1122} & D_{2222} & D_{2212} \\ D_{1112} & D_{2212} & D_{1212} \end{array} \right] \left\{ \begin{array}{c} \varepsilon_{11} \\ \varepsilon_{22} \\ \gamma_{12} \end{array} \right\}, \tag{S1}$$

where σ_{11} and σ_{22} are the normal stress, τ_{12} is the shear stress, D_{1111}, D_{1122}, D_{1112}, D_{2222}, D_{2212} and D_{1212} are the components of the elasticity matrix D, ε_{11} and ε_{22} are the normal strain and γ_{12} is the shear strain. For each component of shear strain, the engineering shear strain γ_{12} , which is twice the amount of the shear component of the strain tensor, is used. By homogenization analysis using XFEM, we obtain D_{1111}, D_{2222} and D_{1212} by applying unit strain in the following three patterns:

$$\left\{ \begin{array}{c} \varepsilon_{11} \\ \varepsilon_{22} \\ \gamma_{12} \end{array} \right\} = \left\{ \begin{array}{c} 1 \\ 0 \\ 0 \end{array} \right\}, \tag{S2a}$$

$$\left\{ \begin{array}{c} \varepsilon_{11} \\ \varepsilon_{22} \\ \gamma_{12} \end{array} \right\} = \left\{ \begin{array}{c} 0 \\ 1 \\ 0 \end{array} \right\}, \tag{S2b}$$

and

$$\left\{ \begin{array}{c} \varepsilon_{11} \\ \varepsilon_{22} \\ \gamma_{12} \end{array} \right\} = \left\{ \begin{array}{c} 0 \\ 0 \\ 1 \end{array} \right\}. \tag{S2c}$$

The mean stress distribution in each direction is written as macroscopic stress in the out-file output of homogenization analysis using XFEM. Since we provide unit strains, D_{1111} is σ_{11} when Eq. (S2a) is substituted into Eq. (S1), D_{2222} is σ_{22} when Eq. (S2b) is substituted into Eq. (S1) and D_{1212} is τ_{12} when Eq. (S2c) is substituted into Eq. (S1).

Owing to the assumption of isothermal forming as the simplest process condition, Young's modulus and Poisson's ratio are expressed using the mean values of D_{1111} and D_{2222}:

$$E = \frac{D_{1212}\left(\frac{3(D_{1111}+D_{2222})}{2} - 4D_{1212}\right)}{\frac{D_{1111}+D_{2222}}{2} - D_{1212}}, \tag{S3}$$

$$\nu = \frac{\frac{D_{1111}+D_{2222}}{2} - 2D_{1212}}{2\left(\frac{D_{1111}+D_{2222}}{2} - D_{1212}\right)}. \tag{S4}$$

Validation of the binarization approach for microstructure images

We discuss the validity of the binarization. Since the microstructures are already binarized and the elasticity matrix D is obtained from the binarized data as input to XFEM, it is sufficient to confirm the validity of the relationship between the crystallization temperature with either the microstructure or the elasticity matrix D. The crystallization temperature used in the phase-field method is before binarization, so we compare the relationship between the microstructures before and after binarization and the crystallization temperature to confirm that the features are not lost.

Due to the limitation of computational resources, it is necessary to compress images when inputting them into machine learning. As Fig. S1 shows, compressing the microstructure images without binarization failed to properly capture the trend of the crystal microstructures. It appears to be particularly difficult to compress images of microstructures in the area near the interface between the crystalline and amorphous phases. Before binarization, the boundary between crystalline and amorphous phases is ambiguous. After binarization, the boundary between crystalline and amorphous phases is clear, and the shape of the crystalline region is similar to that before compression. By performing the binarization, we can make the crystal microstructures before and after the compression look similar, as shown in Fig. S1. During the binarization process, we ensure that the crystal chains are not broken or connected with neighboring crystal chains.

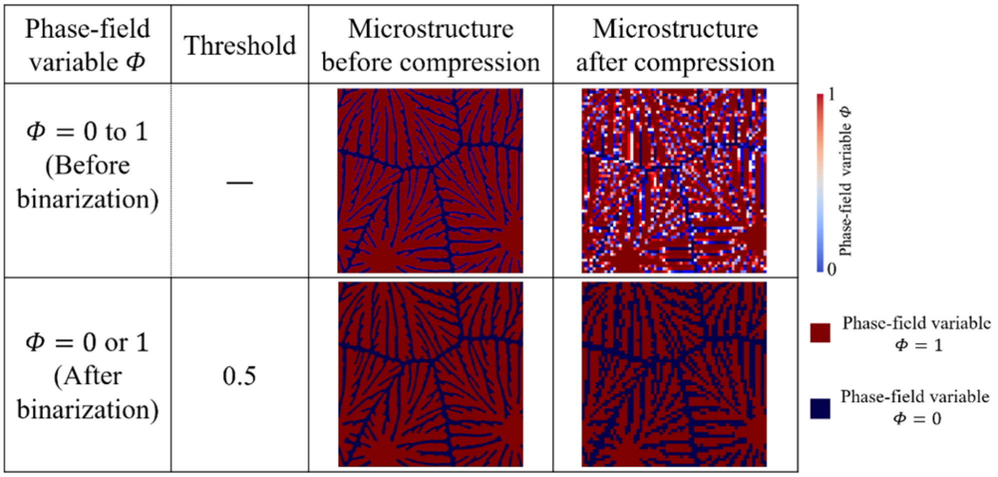

Figure S1. Comparison of compressed microstructures before and after binarization

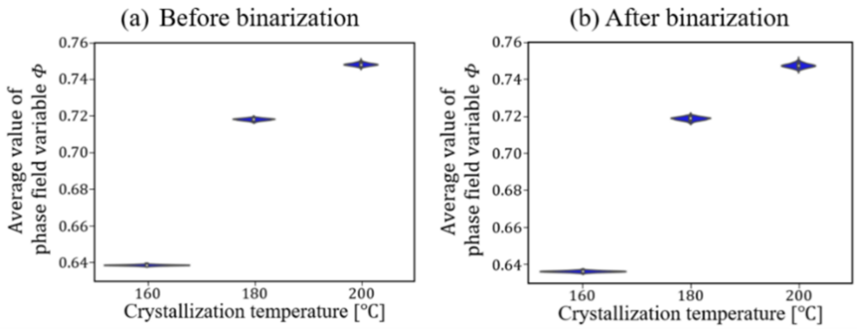

Figure S2. Relationship between crystallization temperature and average value of phase-field variable ϕ (a) before and (b) after binarization

Fig. S2 shows the relationship between the crystallization temperature and the average value of phase-field variable ϕ obtained for the microstructure images before and after binarization. This figure is called a violin plot, and the horizontal axis represents the crystallization temperature and the vertical axis represents the average value of ϕ. As shown in Fig. S2, the relationship between the mean value of ϕ and the crystallization temperature shows a similar trend before and after binarization. The mean value of ϕ increases as the crystallization temperature increases. The magnitude and distribution of the mean value of ϕ remain similar before and after binarization. Fig. S3 shows the results of comparing the average values of ϕ before and after binarization for several images individually. From left to right, the mean value of the phase-field variable ϕ before binarization, the mean value of ϕ after binarization, and the difference between the mean values before and after binarization are shown. The difference shows that there is a slight difference near the interface, but almost no difference otherwise. This implies that the difference has almost no effect on the general shape of the crystalline microstructure. Therefore, binarization is not considered to be a problem.

Validation of microstructure image compression

We check the validity of the compression. Since only the phase-field method uses 320 pixels $\times$ 320 pixels data and the other methods use 64 pixels $\times$ 64 pixels data after compression as training data, it is sufficient to confirm that the trend of crystallization temperature derived from the phase-field method is valid. Figs. S4 and S5 show the relationship between the crystallization temperature specified in the phase-field analysis and the elasticity matrix D obtained by XFEM for the microstructures generated by the phase-field method. The violin plots in Figs. S4 and S5 show the distribution of the data with the horizontal axis for the crystallization temperature and the vertical axis for the value of each component of the elasticity matrix D. Fig. S4 shows training data and Fig. S5 shows test data, (a) is the result of 320 pixels $\times$ 320 pixels microstructures input to XFEM before compression and (b) is the result of 64 pixels $\times$ 64 pixels microstructures input to XFEM after compression. Each violin plot shows that there is a large amount of data in the bulging part of the plot and a large scatter of data in the vertically long part of the plot. The black rectangle in the plot represents the interquartile range of the box plot, and the white

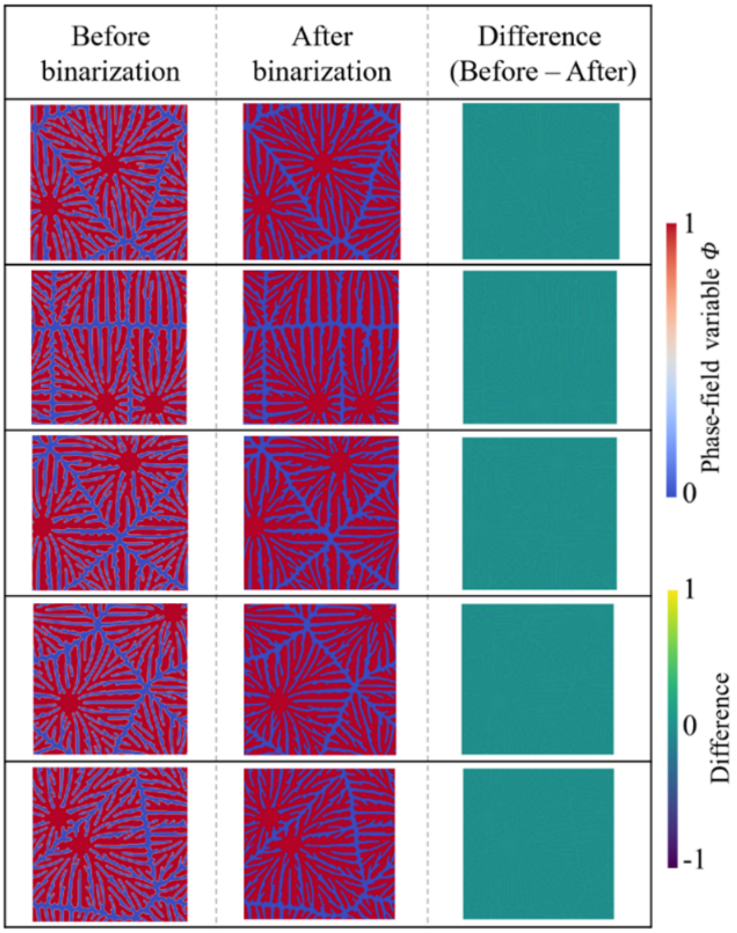

Figure S3. Crystal microstructures before and after binarization and their differences

dot at the center represents the median value. Figs. S4 and S5 show that both before compression (a) and after compression (b), there is a similar trend where higher crystallization temperatures correspond to larger values of the elasticity matrix D. The most frequently occurring value of the the elasticity matrix D increases with higher crystallization temperatures, exhibiting a similar pattern before and after compression. In both Figs. S4 and S5, before compression (a) and after compression (b), D_{1111} and D_{2222} exhibit similar values and distributions, while D_{1212} alone shows smaller values and a narrower distribution compared to the other components, following a similar trend. From the comparison of (a) and (b) in Fig. S4 , and (a) and (b) in Fig. S5, it can be confirmed that the trend of the relationship between crystallization temperature and the components of the elasticity matrix D is similar for both the training and test data, before and after compression. Therefore, it is considered that the compression is not a problem for machine learning data sets.

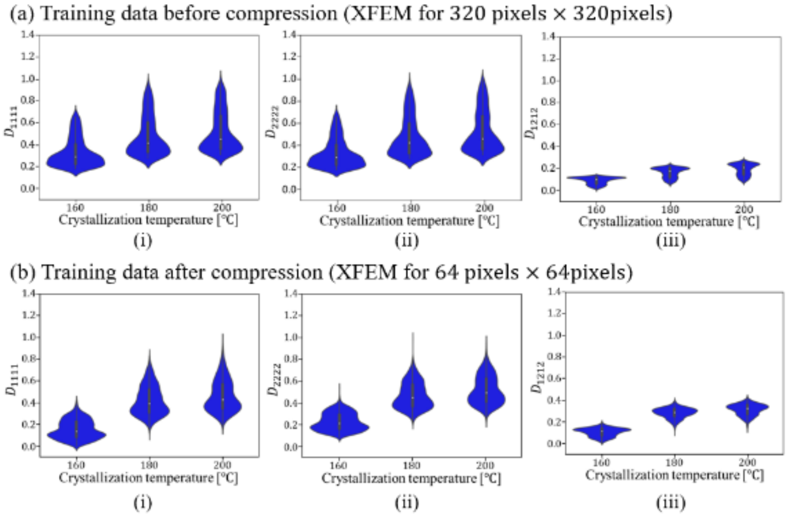

Figure S4. Relationship between crystallization temperature and elasticity matrix (i) D_{1111}, (ii) D_{2222} and (iii) D_{1212} in training data when XFEM input is (a) 320 pixels × 320 pixels image before compression and (b) 64 pixels × 64 pixels image after compression

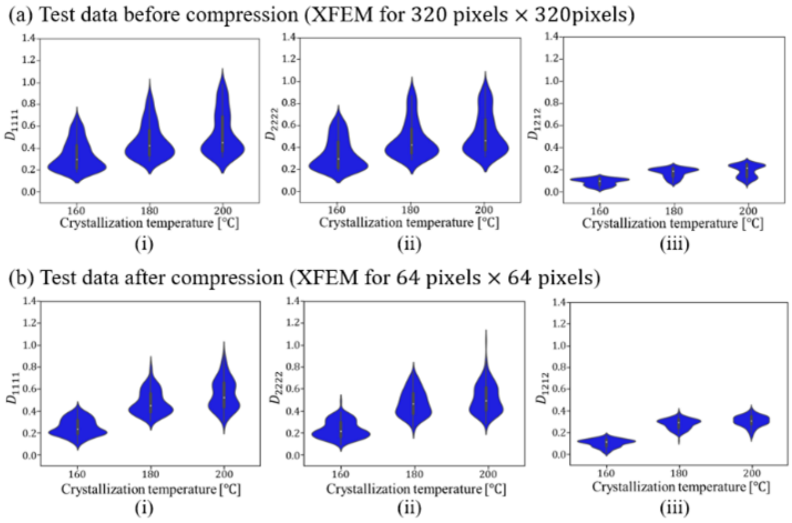

Figure S5. Relationship between crystallization temperature and elasticity matrix (i) D_{1111}, (ii) D_{2222} and (iii) D_{1212} in test data when XFEM input is (a) 320 pixels × 320 pixels image before compression and (b) 64 pixels × 64 pixels image after compression
